# Preoperative Pain, Symptoms, and Psychological Factors related to Higher Acute Pain Trajectories during Hospitalization for Total Knee Arthroplasty

**DOI:** 10.1371/journal.pone.0161681

**Published:** 2016-09-01

**Authors:** Maren Falch Lindberg, Christine Miaskowski, Tone Rustøen, Leiv Arne Rosseland, Steven M. Paul, Anners Lerdal

**Affiliations:** 1 Department of Surgery, Lovisenberg Diakonale Hospital, Oslo, Norway; 2 Department of Nursing Science, Institute of Health and Society, Faculty of Medicine, University of Oslo, Oslo, Norway; 3 School of Nursing, University of California San Francisco, San Francisco, California, United States of America; 4 Department of Research and Development, Division of Emergencies and Critical Care, Oslo University Hospital, Oslo, Norway; 5 Institute of Clinical Medicine, University of Oslo, Oslo, Norway; 6 Department of Patient Safety and Research, Lovisenberg Diakonale Hospital, Oslo, Norway; Université catholique de Louvain, BELGIUM

## Abstract

**Objectives:**

Unrelieved postoperative pain after total knee arthroplasty (TKA) is a significant problem. This longitudinal study investigated how preoperative pain intensity, as well as a comprehensive list of preoperative and perioperative factors, influenced the severity of acute average and worst pain after TKA.

**Methods:**

Prior to surgery, 203 patients completed a demographic questionnaire, Lee Fatigue Scale, Fatigue Severity Scale, Hospital Anxiety and Depression Scale, and Brief Illness Perception Questionnaire. Brief Pain Inventory was completed prior to surgery as well as through postoperative days (POD) 0 to 4. Clinical data were extracted from medical records.

**Results:**

Several factors were associated with higher levels of preoperative and postoperative pain. Lower preoperative average and worst pain intensity scores were associated with increases in average and worst postoperative pain from POD1 to POD4. A higher number of comorbidities, higher C-reactive protein values, and higher pain interference with function were associated with higher preoperative levels of average pain. Older age, higher fatigue levels, and higher scores on identity and emotional responses to osteoarthritis (OA) were associated with higher preoperative levels of worst pain. Lower perceived consequences of OA were associated with higher pain from POD1 to POD4. Males and patients with lower preoperative scores for average pain had higher worst pain following surgery.

**Discussion:**

Patients at higher risk for more severe postoperative pain can be identified through an assessment of pain and other risk factors identified in this study. Future research needs to test the efficacy of interventions that modify patients’ perceptions of living with OA and pain intensity before surgery on short and long term postoperative outcomes.

## Introduction

Approximately 12% of adults over 60 years of age suffer from symptomatic osteoarthritis (OA) of the knee [1]. Living with OA is associated with chronic pain, disability, fatigue, depressed mood, and decreased quality of life [2, 3]. More than 50% of adults diagnosed with knee OA undergo total knee arthroplasty (TKA) [4] in order to relieve pain and improve function. However, TKA is an extremely painful procedure [5–7] and postoperative pain is not well managed [6]. Undertreatment of postoperative pain is associated with a higher risk of pulmonary and cardiac complications [8, 9], delayed recovery, and subsequent development of chronic postsurgical pain [10, 11].

Findings from a systematic review of postoperative pain management [12] noted that additional research is warranted on predictive factors associated with postoperative pain in specific types of surgeries, and should include a comprehensive list of demographic, psychosocial, and surgical characteristics. Knowledge of predictive factors of poorer pain outcomes specific to TKA patients would enable early identification of higher risk patients who warrant more aggressive perioperative pain management.

Higher preoperative pain [13, 14] and preoperative opioid consumption [15] have been identified as risk factors for increased acute pain at rest and with movement after TKA. While preoperative pain, anxiety, and younger age predicted postoperative pain in a variety of surgeries, [12] only one longitudinal study was found that evaluated predictors of acute average and worst pain during hospitalization in patients who underwent TKA [16]. In this study, higher preoperative optimism scores were associated with lower pain intensity scores. In addition, higher presurgical anxiety and worse emotional representations of OA were associated with higher postoperative pain intensity scores. Although a variety of predictors were examined, less than half of the relatively small sample (N = 124) underwent TKA, and results were not reported separately for TKA patients. Therefore, the ability to identify a comprehensive list of risk factors unique for this surgical procedure was limited. Finally, because pain intensity was assessed prior to surgery and only once on postoperative day (POD) 2, the dynamic nature of acute postoperative pain was not evaluated [17]. Chapman [18] stated that the hallmark feature of postoperative pain is systematic change over time and demonstrated how higher measurement precision can be achieved by repeated assessments of acute pain scores over time. Three groups were found with distinct changes in pain during the first six days after various surgeries [18] and cardiac surgery [17]. One group’s pain increased over time, another group’s pain decreased over time, and the third group maintained a constant level of pain. In line with these results, Lavand’Homme [19] found that a subgroup of patients with more severe early pain trajectories reported more pain 3 months after TKA. Our research group have recently reported that higher pain scores with rest and activity on the day of surgery resulted in higher pain trajectories three days following TKA [20]. However, we were unable to find any studies that evaluated the impact of preoperative pain intensity and other potentially modifiable risk factors on acute worst and average pain trajectories during the first week following TKA.

Therefore, to reduce the negative consequences of severe postoperative pain, the purposes of this longitudinal study were to describe the trajectories of average and worst pain intensity using a 0 to 10 numeric rating scale (NRS) from the day of admission (i.e., preoperative pain intensity) until POD 4 and to evaluate how preoperative pain intensity in conjunction with demographic, clinical, symptom, and psychological characteristics, impact inter-individual differences in initial levels of as well as in the trajectories of average and worst postoperative pain (i.e., how pain changes over time). The preoperative pain scores were allowed to be part of the pain trajectories in this study, because we assumed that patients’ preoperative pain levels would impact the course of their acute postoperative pain.

## Materials and Methods

### Patients and study procedures

This study is part of a longitudinal study of pain, symptoms, and health-related quality of life (QOL) in patients who underwent a TKA for OA at a surgical clinic in Oslo, Norway. Patients (n = 203) were included if they were >18 years of age; were able to read, write, and understand Norwegian; and were scheduled for unilateral primary TKA. Patients were excluded if they underwent unicompartmental or revision surgery.

Patients received written information about the study either by mail prior to admission or on the day of admission. Patients were admitted to the hospital between 1 to 3 days prior to surgery, usually on the day before surgery. Patients who met the inclusion criteria were invited to participate by a nurse on the day of admission. After obtaining written informed consent, patients completed a questionnaire that assessed demographic characteristics, preoperative pain, preoperative symptoms, and psychological factors. Preoperative clinical tests, comorbidities, body mass index (BMI), and information on medications were obtained from medical records. The completed questionnaires were collected by the nursing staff in sealed envelopes. The study was approved by the Regional Medical Research Ethics Committee of Health South East of Norway (#2011/1755).

### Preoperative and postoperative pain

The presence and intensity of average and worst preoperative pain was assessed on the day of admission using 0 (no pain) to 10 (worst imaginable pain) NRS. Acute postoperative pain was assessed from the day of surgery (DOS) until POD 4. Patients rated their average and worst pain every evening (i.e., DOS, POD1, POD2, POD3, POD4) using a 0 (no pain) to 10 (worst imaginable pain) NRS. These data were returned to the nursing staff in sealed envelopes.

### Surgical and anesthesiological procedures

The anesthesia, surgery, and postoperative pain management procedures were standardized. All patients received the same posterior cruciate-retaining fixed modular-bearing implant for the TKA. A tourniquet was used during surgery and drains were placed and removed on POD1. Neuraxial block with bupivacaine and sedation were the first choice for anesthesia. Epidural analgesia (EDA), with continuous infusion of bupivacaine 1 milligram/milliliter (mg/ml), adrenaline 2 micrograms (μg)/ml, and fentanyl 2 μg/ml (5 to 12 ml/hour), was used for postoperative pain management. If neuraxial blockade was contraindicated, patients received total intravenous anesthesia and a continuous femoral nerve block (CFNB) with bupivacaine 2.5 mg/ml 4 to 10 ml/hour for postoperative pain management. In most cases, the regional blocks were removed on POD2. Oral acetaminophen 1 gram was given every 6 hours and celecoxib 200 mg and controlled release oxycodone 5 to 20 mg was given every 12 hours unless contraindicated. Immediate release oxycodone 5 mg tablets or intravenous ketobemidone 2.5 to 5 mg were available as rescue medications. If pain control was not satisfactory, low dose ketamine 1.5 μg/kilogram/minute was administered as a short-term intravenous infusion (usually on the DOS).

Mobilization and physical therapy were standardized. All patients were mobilized out of bed and allowed full weight bearing on the operated knee on POD1. Patients received physical therapy on a daily basis with walking, flexion, and extension of the knee beginning on POD1. Most patients were discharged on POD 4.

### Clinical and perioperative characteristics

Data on type of implant, American Society of Anesthesiologists (ASA) physical status classification [21], length of surgery, tourniquet use, infections (i.e., deep prosthetic, wound), as well as comorbidities, BMI, preoperative blood pressure, hemoglobin, C-reactive protein, and creatinine levels were obtained from medical records. Creatinine was included in this analysis because patients with higher levels of creatinine (i.e., males >105, females >90) did not receive celecoxib, which may have led to higher postoperative pain. Higher levels of C-reactive protein and obesity are associated with higher levels of pain in patients with OA but the impact on postoperative pain is not known [22]. Lower resting blood pressure levels are been associated with lower acute pain thresholds [23].

Data on preoperative use of pain medications and sleep medications, anesthesia regimen and doses of postoperative pain medications were obtained from patients’ medical records. For preoperative pain medications and sleep medications, whether a patient used a drug or not (i.e., acetaminophen use, yes/no, opioid use yes/no, sleep medication/benzodiazepine use yes/no), was recorded. According to hospital routines, all except one patient who used a nonsteroidal anti-inflammatory drug discontinued its use 7 to 14 days before admission. For postoperative drug use, all opioid analgesics were converted to intravenous (IV) morphine equivalents using the European Association for Palliative Care recommendations for opioid conversion [24]. Average amount of opioids over the postoperative period was included in the analysis as a covariate, as this variable has the potential to influence postoperative pain.

### Preoperative pain and interference

*The Brief Pain Inventory (BPI)*: Patients’ level of preoperative pain and its impact on function were assessed using the BPI [25]. The BPI consists of four items that measure pain intensity on a 0 to10 NRS; one item that measures pain relief; a body map to assess pain locations; and seven items that measure interference with function. The Norwegian version of the BPI has well-established validity and reliability [26]. Individual items for average and worst pain were used to describe pain severity, based on the IMMPACT recommendations for pain assessment in clinical trials [27].

### Symptom measures

*Fatigue severity*: The 5-item Lee Fatigue Scale (LFS) was used to evaluate preoperative fatigue severity. Each item was rated on a 0 to 10 NRS. A total score was calculated as the mean of the 5 items with higher scores indicating higher fatigue severity. The LFS has satisfactory validity and reliability [28, 29]. In this study, its Cronbach’s alpha was 0.91.

*Fatigue Interference*: The 7-item Fatigue Severity Scale (FSS-7) was used to evaluate fatigue interference during the past week prior to surgery. Patients rated their agreement with 7 statements, using a 7-point Likert scale that ranged from disagree to agree. A total score can range from 1 to 7 with higher scores indicating higher levels of interference. The Norwegian version of the FSS-7 has good psychometric properties [30]. In this study, its Cronbach’s alpha was 0.93.

Fatigue severity and interference were included as potential predictors in the analysis because fatigue and pain occurred together in a symptom cluster in knee and hip osteoarthritis [2, 31]. These two symptoms may share similar underlying pain mechanisms and may also affect the severity of postoperative pain [32].

*The Hospital Anxiety and Depression Scale (HADS)* [33] was used to evaluate depression and anxiety. The scale consists of 14 items (i.e., 7 for depression and 7 for anxiety). On each subscale, scores can range from 0 to 21 with higher scores indicating higher levels of anxiety and depression. Psychometric properties of the Norwegian version of HADS were excellent in a large population-based study in Norway [34]. In this study, the Cronbach’s alphas for the depression and the anxiety scales were 0.84 and 0.79, respectively.

### Psychological measures

*The Brief Illness Perception Questionnaire (BIPQ)*: The BIPQ [35] was used to measure self-reported illness perceptions. This questionnaire is based on Leventhal and colleagues’ self-regulatory model that describes the process by which individuals respond to a perceived health threat. The scale consists of eight items that measure different dimensions of self-reported illness perception (i.e., consequences, timeline, personal control, treatment control, identity, illness concern, coherence, and emotional response). Each item was rated on a 0 to 10 NRS. For this study, the patients rated their illness perception in relation to their OA knee. Five items from the BIPQ (i.e., consequences, personal control, identity, concern, emotional response) were used in the statistical analyses because these specific items were sensitive to changes over time in patients with traumatic injuries [36]. For consequences, patents rated how much their OA affected their lifes, with the endpoints 0 (no affect at all) to 10 (severely affects my life). For personal control, patients rated how much control they felt they had over their OA, with the endpoints 0 (absolutely no control) to 10 (extreme amount of control). For identity, patients rated how much they experienced symptoms from their OA, with the endpoints 0 (no symptoms at all) to 10 (many severe symptoms). For concern, patients rated how concerned they were about their illness, with the endpoints 0 (not at all concerned) to 10 (extremely concerned). For emotional response, patients rated how much their illness affected them emotionally (e.g., made them angry, scared, upset or depressed), with the endpoints 0 (not at all affected emotionally) to 10 (extremely affected emotionally).

### Statistical analyses

Descriptive statistics and frequency distributions were performed on the preoperative demographic, clinical, symptom, and psychological characteristics of the sample using SPSS version 22 (IBM, Armonk, NY). The distributions of the pain intensity scores were consistently symmetrical, used the full range of values from 0 to 10, and were not generally skewed either to the right or the left at any of the six measurement times. In addition, our sample size was large enough to provide confidence that the assumptions of the statistical models were not violated. Power analyses specific for all of the different elements of the HLM analyses were not performed. A sample of 203 patients provides power of at least 80%, at an alpha of .05, to detect as significant a correlation as small as r = .19. From a multiple regression point of view, a sample of 203 patients provides power of at least 80%, at an alpha of .05, to detect as significant an overall percent of explained variance (R^2^) of 6.8% from a model with seven predictors. A R^2^ of 2% is considered a small effect. A R^2^ of 13% is considered a medium effect. A R^2^ of 26% is considered a large effect [37]. The sample of 203 patients provided adequate power to detect small to medium effect sizes. Hierarchical linear modeling (HLM) based on full maximum likelihood estimation, was performed in two stages using the HLM 6 software developed by Raudenbush and Bryk [38]. Previous publications have discussed this analysis in detail [39–44]. In brief, intra-individual variability in pain intensity (i.e., average pain, worst pain) over time was investigated in stage 1. At this point, the model was constrained to be unconditional (i.e., no predictors) and likelihood ratio tests were used to determine the best model. To evaluate the pattern of change in pain intensity, a piecewise model strategy was used, as the assessment period included both preoperative and postoperative measurements. Therefore, the six assessments were coded into two pieces. Piece 1 (PW1) consists of assessment 1 and 2, which were used to model changes over time from before surgery until the day of surgery. Piece 2 (PW2) consists of assessments 3 through 6 which were used to model changes over time from POD1 to POD4. A piecewise model can be more sensitive to the timing and sequencing of changes in a dependent variable than conventional HLM models, which would have assessed changes over the six assessments without taking into account the two different pre- and postoperative stages.

The second level of the HLM analysis examined inter-individual differences in the piecewise trajectories of postoperative pain intensity by modeling the individual change parameters (i.e., intercept and slope parameters) as a function of proposed predictors. Table 1 presents a list of the proposed predictors that was developed based on a qualitative review of the literature on factors associated with perioperative pain intensity and acute pain in patients undergoing TKA [12, 14–16, 45, 46].

**Table 1:** Potential predictors of intercept (I), piecewice 1 (PW1), piecewice 2 (PW2), linear (L), quadratic (Q) and cubic (C) components for average and worst pain.

	Average pain					Worst pain				
Demographic characteristics	I	PW1 L	PW2 L	PW2 Q	PW2 C	I	PW1 L	PW2 L	PW2 Q	PW2 C
Age						x				
Sex	x						x			
Education level										
Cohabitation status										
Employment status						x				
**Preoperative clinical characteristics**										
Body mass index		x								
Number of comorbidities	x					x				
American Society of Anesthesiologists’ physical status classification										
Systolic blood pressure						x				
Diastolic blood pressure										
C-reactive protein	x									
Hemoglobin										
**Preoperative pain characteristics**										
Preoperative use of acetaminophene										
Preoperative use of opioids	x		x							
Preoperative sleep medication/benzodiazepines										
Average pain prior to surgery	n/a		x	x	x	n/a	x			
Worst pain prior to surgery						n/a		x	x	x
Pain interference with function prior to surgery	x					x	x	x	x	x
**Perioperative characteristics**										
Side of knee surgery	n/a	n/a				n/a	n/a			
Type of anesthesia	n/a	n/a	x	x	x	n/a	n/a	x	x	
Length of surgery (minutes)	n/a	n/a				n/a	n/a			
**Pain management characteristics**										
Number of days with epidural analgesia*	n/a	n/a	x	x	x	n/a	n/a	x	x	
Number of days with continuous femoral block*	n/a	n/a	x	x	x	n/a	n/a	x	x	
Number of days with ketamine *	n/a	n/a	x	x	x	n/a	n/a	x		
Average dose of opioids over 4 days*	n/a	n/a	x	x	x	n/a	n/a			
**Symptoms**										
Fatigue severity	x					x				
Fatigue interference	x					x				
Depression	x					x				
Anxiety	x					x				
**Psychological characteristics from the Brief Illness Perception questionnaire**										
Consequences	x	x				x				x
Personal control						x				
Identity	x					x				
Concern	x					x				
Emotional response	x					x				

X indicates *t*-values higher than 2.0 in exploratory analyses.

*Variables included in analysis as covariates

To improve estimation efficiency and construct a parsimonious model, exploratory level 2 analyses were performed. Each potential predictor was assessed to determine whether it would result in a better fitting model if it alone was added as a level 2-predictor. Predictors with a *t* value of <2.0, which indicates a lack of significant effect, were excluded from further model testing. All potential significant predictors from the exploratory analysis were entered into the model to predict each individual change parameter. Only predictors that maintained a statistically significant contribution in conjunction with other variables were retained in the final model. A p-value of < .05 indicated statistical significance. The unique contribution of each significant predictor on the pain trajectories for patients’ with higher versus lower scores on the predictor variable, while controlling for all other variables in the model are shown in figures to provide the reader with the clinical meaning of each predictor variable. Effect sizes (ES) were calculated on the differences in pain scores for patients with high versus low scores on a predictor variable, according to Cohen’s coefficient *d* (i.e., Small = >.2; Medium = >.5; Large = >.8). A *d* -value ≥0.40 was considered a clinically meaningful difference between groups [47].

## Results

Of the 245 patients invited to participate, 6 had their surgery canceled and 33 declined participation. A total of 206 patients (84%) were enrolled in the study. Two patients were excluded after surgery because of postoperative disorientation and one patient died from postoperative complications. Of the 206 who were enrolled, 203 (98.5%) patients are included in this analysis.

Table 2 summarizes the demographic, clinical, symptom, and psychological characteristics of the patients. The sample was predominantly female (69%), with a mean age of 68 (± 9.2) years, were living with a partner (61%), were currently not working (66%), and half of the patients (50%) had completed higher education.

**Table 2:** Demographic, clinical, symptom, and psychological characteristics of patients (N = 203) prior to surgery.

Characteristic				
**Demographic characteristics**			Mean	SD
Age	Years		68.2	9.2
			n	%
Sex	Female		139	68.5
Cohabitation status	Married/partnered		123	60,6
Employment status	Unemployed/retired		131	64.5
Education level	College/university		102	51.0
**Preoperative clinical characteristics**			Mean	SD
	Body mass index		29.2	4.8
		Median		
	Number of comorbidities (0–5)	1.0	1.2	1.0
	American Society of Anesthesiologists’ physical status classification score (1–3)	2.0	2.0	0.5
	Systolic blood pressure		137.9	16.1
	Diastolic blood pressure		81.2	11.4
	C-reactive protein		3.2	3.0
	Hemoglobin		13.8	1.1
	Creatinine		76.5	21.9
**Preoperative pain characteristics**			Mean	SD
	Average preoperative pain (0–10)		5.3	1.8
	Worst preoperative pain (0–10)		5.5	2.1
	Pain interference with function (0–10)		4.4	2.0
			n	%
	Preoperative use of acetaminophen		42	20.7
	Preoperative use of opioids		18	8.9
	Preoperative use of sleep medication/benzodiazepines		32	15.8
**Perioperative characteristics:**				
Surgical side	Right side		104	51.2
Anesthesia	Neuraxial block		176	86.7
	Total intravenous anesthesia		27	13.3
			Mean	SD
	Length of surgery (minutes)		65.38	13.5
**Pain management characteristics**		Median		
	Number of days with epidural analgesia (n = 173, 0–3)	2.0	2.1	0.4
	Number of days with continuous femoral block (n = 31, 0–3)	2.0	2.1	0.4
	Number of days with ketamine (n = 30, 0–2)	1.0	1.3	0.5
	Average dose of opioids (0–68 mg)*	11.9	13.0	7.4
	Opioid consumption day of surgery (0–80.5 mg)	7.5	12.5	13.6
	Opioid consumption postoperative day 1 (0–75.5 mg)	12.5	13.4	8.9
	Opioid consumption postoperative day 2 (0–55 mg)	15.0	15.6	8.0
	Opioid consumption postoperative day 3 (0–60.5)	10.0	10.7	8.5
**Symptoms**			Mean	SD
	Fatigue severity (1–10)		2.7	2.1
	Fatigue interference (1–7)		3.9	1.5
	Depression (0–21)		3.5	3.1
	Anxiety (0–21)		4.6	3.5
Psychological characteristics**				
	Consequences (0–10)		6.3	1.8
	Personal control (0–10)		5.4	2.4
	Identity (0–10)		6.6	1.7
	Concern (0–10)		5.0	2.6
	Emotional response (0–10)		4.5	2.6

*All opioids were converted to intravenous morphine equivalents. Value is the average dose of opioids over 4 days.

**Single item scores from the Brief Illness Perception Questionnaire

### Unconditional changes in pain intensity over time

The first HLM analysis examined how pain intensity scores changed within PW1 (i.e., pain intensity prior to surgery until DOS) and within PW2 (i.e., POD1 through POD4). For both average and worst pain, the goodness of fit tests for the deviance among the models indicated that a linear fit for PW1 and a cubic fit for PW2 was the best. The estimates for the initial piecewise models are presented in Table 3. Since the models have no covariates, the intercepts represent the estimated levels of average (5.286) and worst (5.512) pain intensity prior to surgery.

**Table 3:** Hierarchical linear models of the trajectories for average and worst postoperative pain.

**Average pain (n = 203)**	**Coefficient (SE)**	
	Unconditional model	Final model
**Fixed effects**		
Intercept	5.286 (0.133)***	5.284 (0.122)***
Piecewise 1—linear rate of change	-2.682(0.158)***	-2.682 (0.153)***
Piecewise 2 –linear rate of change	1.629 (0.298)***	1.568 (0.290)***
Piecewise 2 –quadratic rate of change	-0.858 (0.192)***	-0.827 (0.187)***
Piecewise 2 –cubic rate of change	0.130 (0.032)***	0.126 (0.031)***
Time invariant covariates		
Intercept:		
Number of comorbidities		0.213 (0.079)**
C-reactive protein		0.062 (0.026)*
Pain interference with function		0.362 (0.047)***
Piecewise 1—linear rate of change		
Consequences		-0.242 (0.051)***
Piecewise 2—linear rate of change		
Average dose of opioids+		0.099 (0.033)**
Average preoperative pain		-0.323 (0.146)*
Piecewise 2—quadratic rate of change		
Average dose of opioids+		-0.040 (0.023)
Average preoperative pain		0.194 (0.102)
Piecewise 2 –cubic rate of change		
Average dose of opioids+		0.004 (0.004)
Average preoperative pain		-0.028 (0.017)
**Variance components**		
In intercept	1.168***	0.734***
Goodness of fit deviance (df)	4145.191 (7)	4033.050 (17)
Model comparison χ^2^ (df)		112.141 (10)***
**Worst pain (n = 203)**	**Coefficient (SE)**	
	**Unconditional model**	**Final model**
**Fixed effects**		
Intercept	5.512 (0.174)***	5.512 (0.164)***
Piecewise 1—linear rate of change	-1.279 (0.212)***	-1.295 (0.205)***
Piecewise 2 –linear rate of change	1.975 (0.404)***	1.902 (0.391)***
Piecewise 2—quadratic rate of change	-0.852 (0.259)**	-0.817 (0.251)**
Piecewise 2 –cubic rate of change	0.101 (0.043)*	0.097 (0.041)*
Time invariant covariates		
Intercept:		
Age		-0.026 (0.012)*
Fatigue severity		0.206 (0.057)***
Identity		0.253 (0.076)**
Emotional response		0.189 (0.047)***
Piecewise 1—linear rate of change		
Sex (men as reference)		-0.475 (0.228)*
Average preoperative pain		-0.216 (0.068)**
Piecewise 2—linear rate of change		
Worst preoperative pain		-0.603 (0.171)***
Piecewise 2 –quadratic rate of change		
Worst preoperative pain		0.343 (0.117)**
Piecewise 2 –cubic rate of change		
Worst preoperative pain		-0.050 (0.020)*
**Variance component**		
In intercept	1.810***	1.374***
Goodness of fit deviance (df)	4792.618 (7)	4695.011 (16)
Model comparison χ^2^ (df)		97.607 (9)***

Note: Time was coded as zero on the day of admission

Intercept = day of admission. Piecewice 1 = day of admission to DOS. Piecewice 2 = POD1 to POD4

+ Variable included in analysis as a covariate

*p < .05

**p < .01

***p < .001

Abbreviations: df = degrees of freedom; SE = standard error

**Fig 1:**
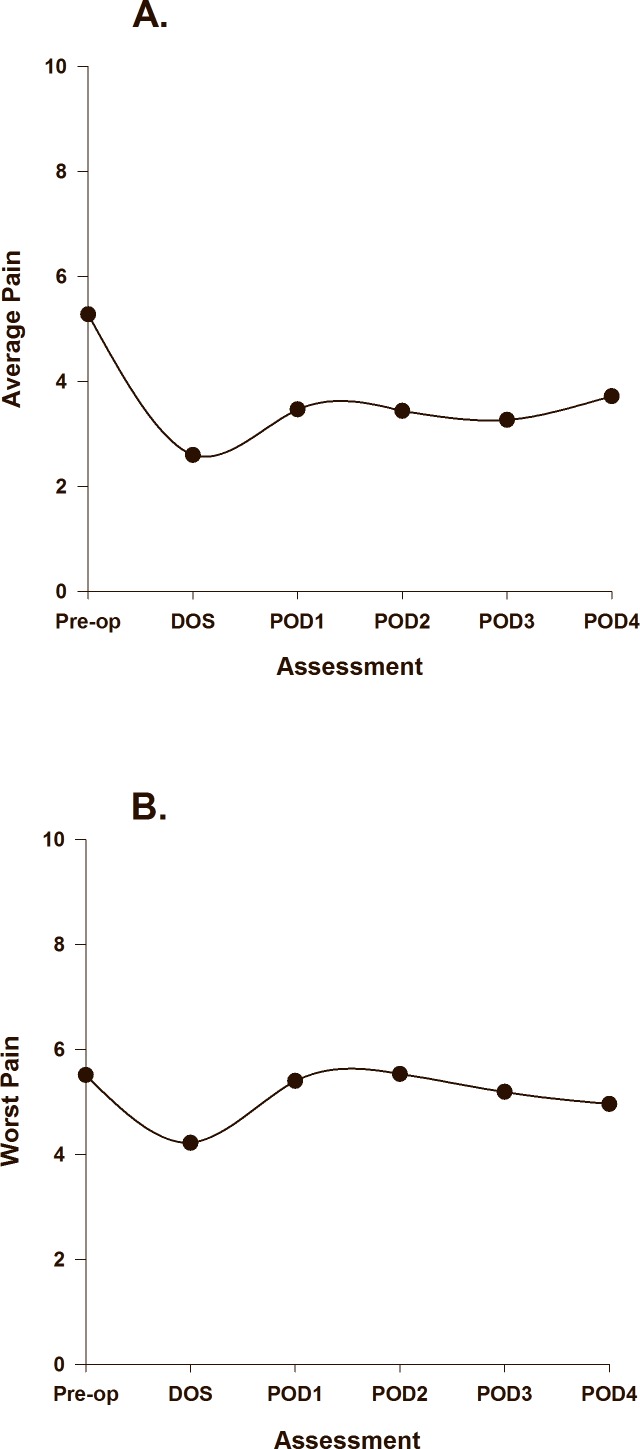
Trajectories of average pain (A), and worst pain (B) using an unconditional model.

The combination of each of the coefficients in the unconditional models determines the slope of the curves for each of the piecewise components’ changes in average and worst pain over time. The unconditional estimated scores for changes in average and worst pain over time are based on the predicted values from the HLM analysis and are illustrated in Fig 1A and 1B, respectively. The equations for the unconditional models for pain could be represented as: Predicted Pain = ß0 + ß1(PW1) + ß2(PW2) + ß3(PW2^2^) + ß4(PW2^3^). The different combination of values of PW1 and PW2 for each of the six assessment days can be put into the equation yielding a different predicted value for each day. Utilizing the regression coefficients found in Table 3 generates the following equations for the unconditional models of average and worst pain: Predicted Average Pain = 5.286 + -2.682(PW1) + 1.629(PW2) + -0.858(PW2^2^) + 0.130(PW2^3^). Predicted Worst Pain = 5.512 + -1.279(PW1) + 1.975(PW2) + -0.852(PW2^2^) + 0.101(PW2^3^).

With no covariates in the model, average pain decreased significantly from prior to surgery to the DOS, increased on POD1 and remained relatively stable through POD2 and POD3, and then increased slightly on POD4. The average postoperative pain intensity scores never exceeded the level of average pain prior to surgery. With no covariates in the model, worst pain decreased from prior to surgery to the DOS; increased on POD1 reaching a peak on POD2 that slightly exceeded preoperative scores, followed by a slight decrease on POD4.

### Inter-individual differences in the trajectories of average pain

**Fig 2:**
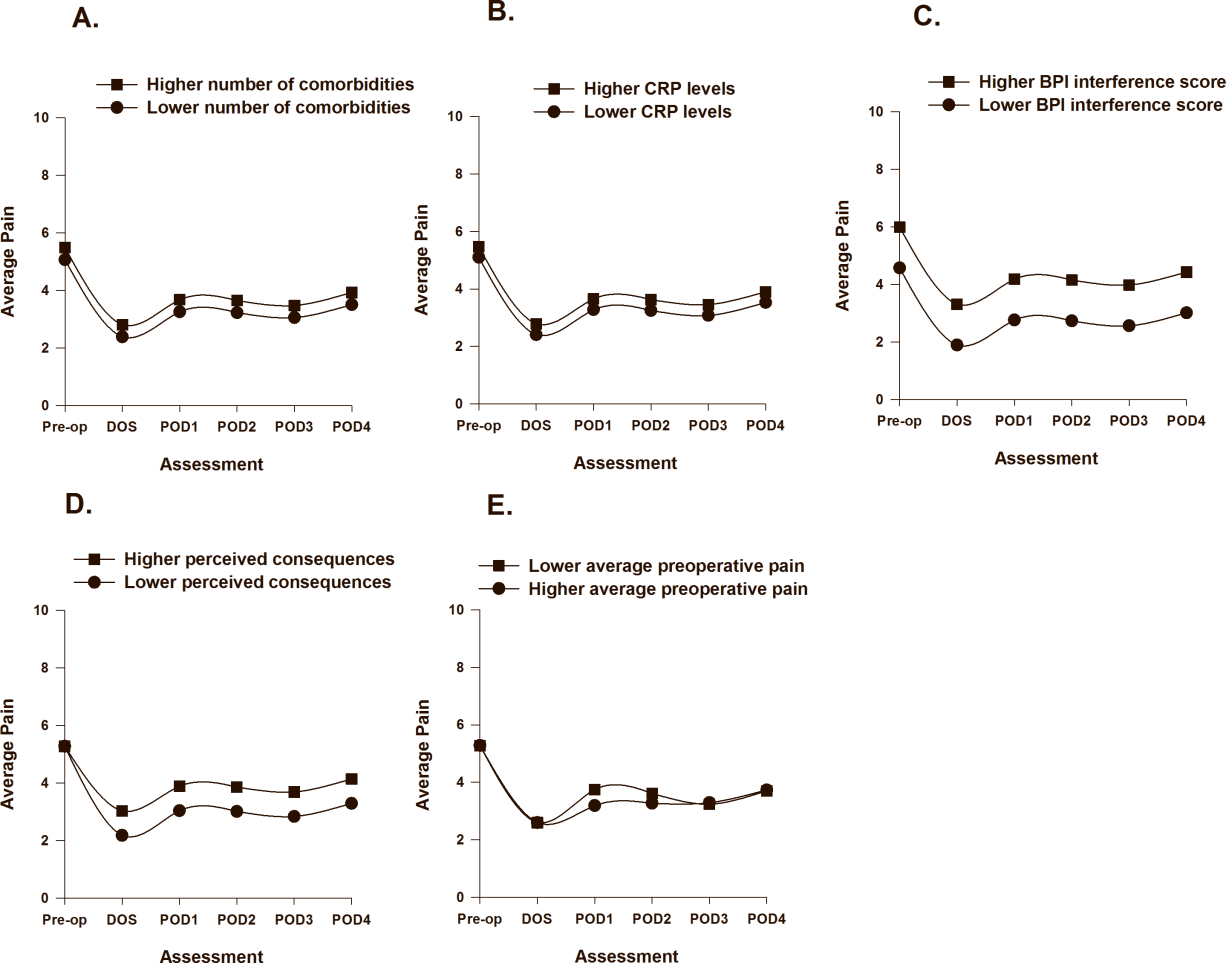
Trajectories of average pain by number of comorbidities (A), CRP levels (B), BPI interference scores (C), perceived consequences of osteoarthritis (D), and average preoperative pain intensity (E) from before surgery until postoperative day 4. Higher/lower differences in Fig 2 A to F were calculated based on 1 standard deviation above/below the mean. The coefficients are adjusted for all other variables in the model.

As displayed in Table 3, two clinical (i.e., number of comorbidities, C-reactive protein) and one symptom (i.e., pain interference with function) characteristics were associated with initial levels (i.e., intercept, preoperative pain intensity) of average pain. The PW1 slope was associated with one psychological (i.e., consequences) characteristic and the PW2 slope was associated with one perioperative covariate (i.e., average dose of opioids) and one symptom (i.e., average preoperative pain intensity) characteristic. The impact of number of comorbidities (Fig 2A), C-reactive protein (2B), pain interference (2C), consequences (Fig 2D), and average postoperative pain (Fig 2E) are illustrated based on two groups scoring ± 1 SD on each of the predictor variables.

While average dose of opioids and average preoperative pain intensity only made significant contributions to parts of the change in average pain intensity over time, they improved the overall model fit, and findings from previous studies suggest that they are important predictors of postoperative pain [13, 14, 48]. Average preoperative pain was a variable of particular interest in this study. A partly significant predictor may still explain some of the variance in the model. Thus, these variables were retained in the final model

### Inter-individual differences in the trajectories of worst pain

**Fig 3:**
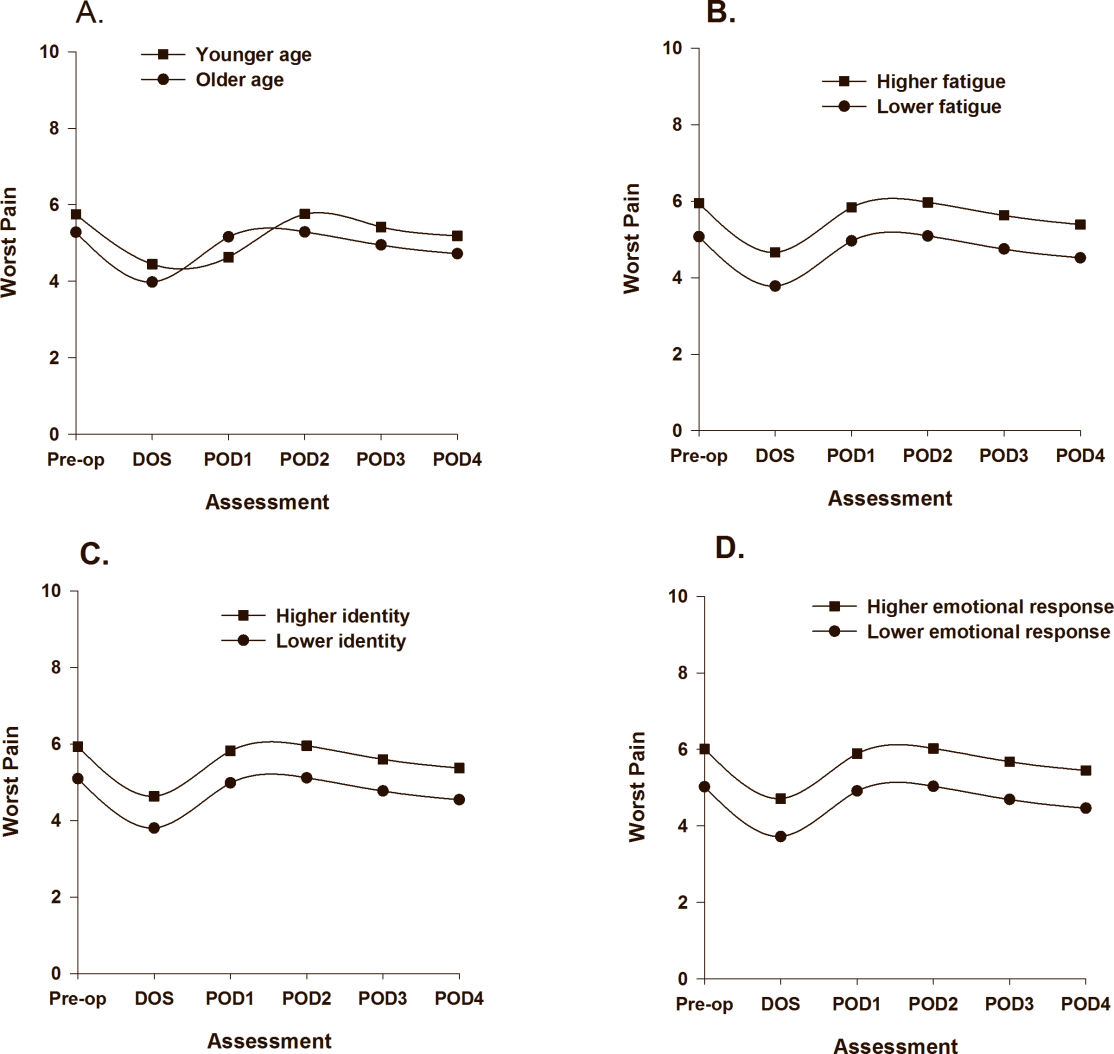
Trajectories of worst pain by age (A), fatigue (B), identity scores (C), and emotional response of osteoarthritis (D) frombefore surgery until postoperative day 4. Higher/lower differences in Fig 3 B to D were calculated based on 1 standard deviation above/below the mean. The coefficients are adjusted for all other variables in the model.

**Fig 4:**
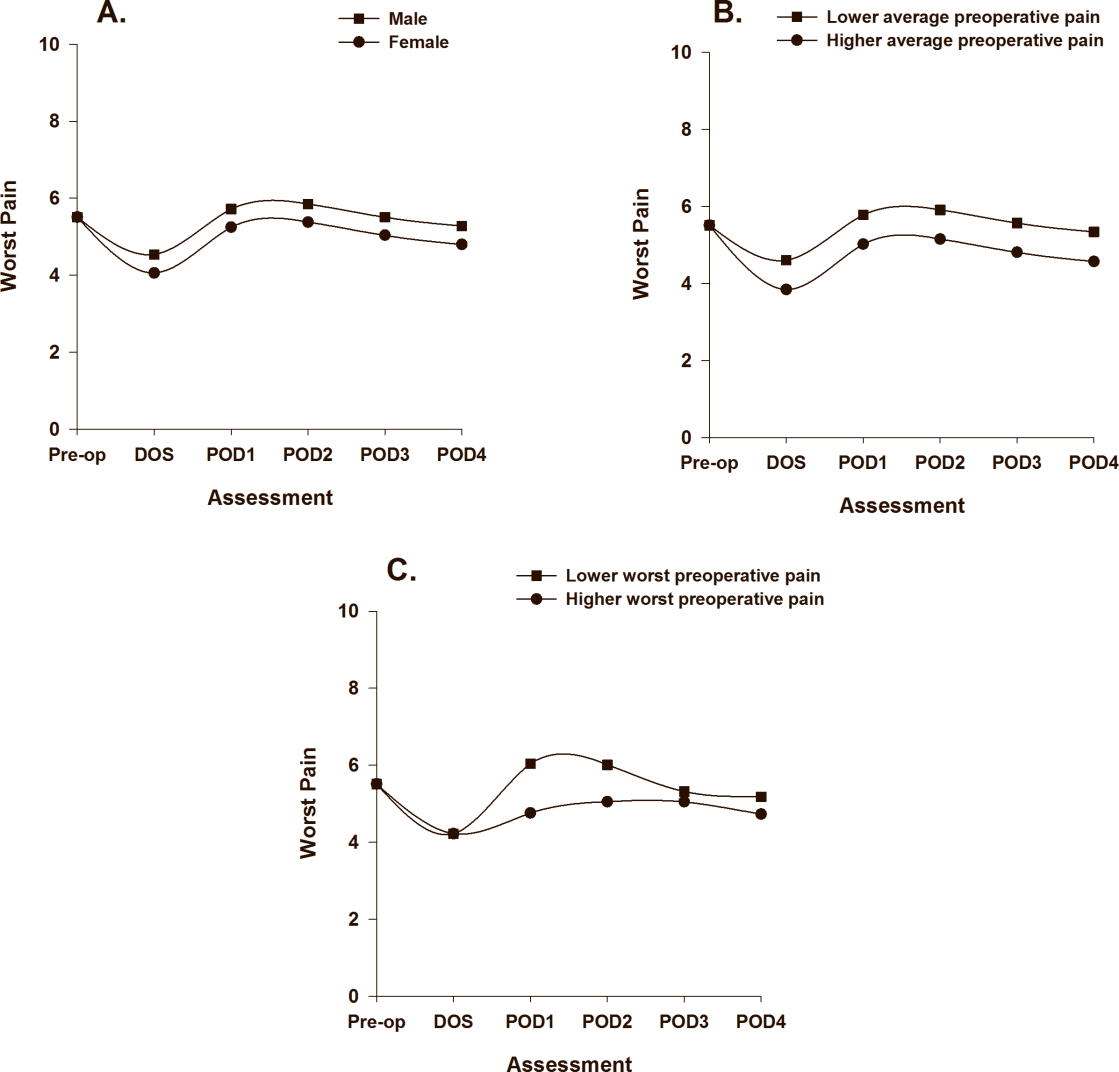
Trajectories of worst pain by gender (A), average preoperative pain intensity prior to surgery (B), and worst preoperative pain intensity (C) from before surgery until postoperative day 4. Higher/lower differences in Fig 4 B to C were calculated based on 1 standard deviation above/below the mean. The coefficients are adjusted for all other variables in the model.

As displayed in Table 3, one demographic (i.e., age), one symptom (i.e., LFS score), and two psychological (i.e., identity, emotional response) characteristics were associated with initial levels (i.e., intercept, preoperative pain intensity) of worst pain. The PW1 slope was associated with one demographic (i.e., gender) and one symptom (i.e., average preoperative pain intensity prior to surgery) characteristic. The PW2 slope was associated with one symptom (i.e., worst preoperative pain intensity scores prior to surgery) characteristic. The impact of age (Fig 3A), LFS score (Fig 3B), identity (Fig 3C), and emotional response (Fig 3D) on initial levels of worst pain trajectories are illustrated based on two groups scoring ± 1 SD on each of the intercept predictors. The impact of gender (Fig 4A), average preoperative pain intensity (Fig 4B), and worst preoperative pain intensity (Fig 4C) on the slopes of worst pain are illustrated based on scores for males/females, and based on two groups scoring ± 1 SD on preoperative average and worst pain.

## Discussion

This study is the first to examine the effects of a comprehensive list of demographic, clinical, symptom, and psychological predictors of changes in average and worst postoperative pain over the first four days following TKA. Our analytic method, complementary to previous studies using linear or logistic regression models, allowed us to evaluate more complex pain trajectories during the first four postoperative days as well as the relationships between different predictors throughout the immediate postoperative period.

For both average and worst pain over time, a distinct increase in pain intensity occurred on POD2, which coincides with the removal of nerve blocks as well as increases in activity levels. For average pain, all of the postoperative pain scores were lower than the preoperative pain scores, with the highest postoperative score occurring on POD4. This finding may be the result of the intensive postoperative pain management regimen that these patients received. However, for worst pain, the trend was somewhat different. Worst pain scores slightly exceeded the preoperative pain ratings on POD2 and gradually declined until POD4. These findings suggest that the measurement of worst pain might capture different aspects of the pain experience such as elements of dynamic or movement evoked pain. The relatively similar levels of average and worst pain prior to surgery may be attributed to reduced activity levels as a consequence of pain and disability associated with OA of the knee [49].

Several characteristics were identified that may assist clinicians to identify patients at higher risk for increased pain during hospitalization. Lower average and worst preoperative pain intensity scores were associated with slight increases in average and worst postoperative pain intensity scores from POD1 to POD3 (Figs 2E and 4C). In addition, consistent with a previous report [50], lower average preoperative pain intensity was associated with slower decrease in worst pain from prior to surgery until the DOS (Fig 4B). However, in recent studies on acute pain after TKA that used regression analysis, higher preoperative pain was associated with higher postoperative pain with rest and movement [13, 14]. In the current study, for both average and worst postoperative pain, the PW1 slope represents the transition from preoperative to acute postoperative pain as well as the transition to a multimodal pain management plan that was initiated on the DOS. Our results suggest that patients with higher preoperative pain scores have more to gain in terms of pain relief. Alternatively, they may have adapted to their OA pain condition and had more realistic expectations about pain management in the postoperative period [51].

Not surprisingly, patients with higher preoperative pain interference with function had higher average pain scores prior to surgery (Cohen’s *d* = 0.8). In addion, patients with higher levels of preoperative CRP had higher average preoperative pain. While the effect size for CRP was small (Cohen’s *d* = 0.2), it is interesting to note that the variance in serum levels was relatively small (range 1 to 20 mg/l), which suggests that even small variations in CRP are associated with higher levels of preoperative pain. In a recent systematic review that evaluated for associations between serum levels of CRP and pain, function, and radiographic changes in OA patients compared to healthy controls [22], weak but significant correlations were found between higher CRP levels and higher pain scores as well as decreases in physical function. No correlation was found between CRP levels and radiographic changes, which suggest that low grade systemic inflammation may play a role in patients’ symptom experiences but not in cartilage deterioration.

Patients with a higher emotional response to OA (Cohen’s *d* = 0.5), as well as patients attributing more symptoms to their OA (Cohen’s *d* = 0.4) reported higher worst preoperative pain scores (Fig 3C and 3D). This finding is consistent with a previous study that found that higher emotional representations of OA prior to surgery were associated with higher postoperative pain 48 hours after TKA or hip replacement [16]. In contrast, patients in our study who perceived that their OA had less consequences for their lives had slower decreases in ratings of average pain following surgery (Fig 2D) (Cohen’s *d* = 0.5). Since lower perceived consequences of OA were associated with lower average pain scores (r = -.49, p < .001) and lower pain interference with function (r = -.64, p < .001) prior to surgery, these patients may have been surprised by the severity of the pain and associated disability following TKA. Illness perceptions are defined as beliefs [35] and are considered modifiable. Preoperative education may provide patients with more realistic expectations about postoperative pain and disability [51]. In previous studies, written information aimed at modifying illness perceptions improved patients’ attendance at rehabilitation [52], their understanding of their illness, and accelerated their recovery and return to work [53]. However, it is not known if modifying patients’ illness perceptions will have an impact on postoperative pain outcomes following TKA.

Consistent with previous studies [14, 46], younger age was associated with higher worst pain scores following surgery. While women reported higher worst preoperative pain intensity scores (5.71, SD 2.0) compared to men (5.09, SD 2.2), being female was associated with faster improvement in worst pain from prior to surgery until the DOS (Cohen’s *d* = 0.7). However, worst pain scores for women and men followed a similar trend from POD1 to POD4 (Fig 4A). Several plausible explanations for these gender differences exist. Since women have smaller bodies than men, the standardized doses of oral analgesics used in this study may have resulted in higher postoperative plasma concentrations in women [54]. In addition, while no statistically significant differences in opioid consumption were found between men and women (p = 0.13), previous research demonstrated that opioid receptor agonists are more efficacious in women [54]. Similar patterns were found in a large study of gender differences in pain trajectories following a variety of surgical procedures [18]. Women may also have more realistic expectations about postoperative pain and disability [51] prior to TKA. Finally, women’s pain is more likely to be assessed accurately by clinicians [55]. Our findings suggest that clinicians may need to perform more detailed pain assessments of men following TKA.

A higher number of comorbidities was associated with higher average preoperative pain intensity. The associations between higher levels of comorbidity and increased pain and poorer function are well known [56, 57]. While the effect size for comorbidities on pain was small (Cohen’s *d* = 0.24), clinicians need to take this risk factor into consideration when they individualize a patient’s pain management plan.

Some study limitations warrants consideration. The majority of the sample was female, which is reflective of the OA population [58]. Patients were recruited from one single surgical clinic, which may limit the generalizability of our findings. In addition, we did not collect data on the use of rescue medication and patients’ ability to perform physical therapy. A number of statistical comparisons were made which may have increased the risk for type 1 error. Because of the exploratory nature of this study, each independent variable was evaluated against an alpha level of 0.05, but most of the variables included in the models have p-values of less than .001. Finally, patients in this study received a comprehensive and individualized postoperative pain management regimen. Therefore, a number of factors related to the pain management regimen were included into our analysis. While reflective of the complexity of clinical reality, these factors may have impacted our results.

The study has several strengths. First, this study evaluated a comprehensive list of predictors using novel statistical methods. Secondly, the sample size is relatively large with a minimal amount of missing data. Third, only patients who underwent TKA for OA and received the same implant were included. Fourth, patients with a wide age range were included, which increases the generalizability of our findings. Older patients tend to be excluded from these types of studies [12].

In conclusion, we found that patients with lower average and worst preoperative pain scores had higher postoperative pain over time. Possible modifiable predictors such as perception of OA illness and pre-operative pain intensity were identified. Clinicians may use these factors to identify patients at higher risk for more severe postoperative pain.Future research should focus on the development of a screening tool to identify patients at higher risk and an evaluation of the effects of interventions that modify these risk factors on short and long term pain interference, function and quality of life.

## Supporting Information

S1 Table Overview of significant predictors of intercept, piece 1, and piece 2 for average and worst pain trajectories. (DOCX)
