# Supplementary material for: Preoperative Pain, Symptoms, and Psychological Factors related to Higher Acute Pain Trajectories during Hospitalization for Total Knee Arthroplasty
Source: PLoS One. 2016 Sep 1;11(9):e0161681. doi: 10.1371/journal.pone.0161681 (PMC5008744; doi:10.1371/journal.pone.0161681)
Supplement: S1 Table — (DOCX) [file pone.0161681.s001.docx]

**S1 Table. Overview of significant predictors of intercept, piece 1, and piece 2 for average and worst pain trajectories**

| **Predictors** | **Average pain** | **Worst pain** |
| --- | --- | --- |
| Age |  | I |
| Sex |  | PW1 |
| Number of comorbidities | I |  |
| C-reactive protein | I |  |
| Average preoperative pain | PW2 | PW1 |
| Worst preoperative pain |  | PW2 |
| Pain interference with function | I |  |
| Average dose of opioids* | PW2 |  |
| Fatigue interference |  | I |
| Consequences | PW1 |  |
| Identity |  | I |
| Emotional response |  | I |

Abbreviations: I = intercept, PW1= piece 1, PW2 = piece 2
*Variable included in analysis as a covariate
